# Response to “Treatment compliance and effectiveness in complex PTSD patients with co-morbid personality disorder undergoing stabilizing cognitive behavioral group treatment: a preliminary study”

**DOI:** 10.3402/ejpt.v5.23489

**Published:** 2014-02-05

**Authors:** Ad De Jongh, Erik Ten Broeke

**Affiliations:** 1 Department of Behavioral Sciences, ACTA, University of Amsterdam and VU University, Amsterdam, The Netherlands, School of Health Sciences, Salford University, Manchester, United Kingdom; 2 Visie, Private Practice for Cognitive Behavioural Therapy, Deventer, The Netherlands

Last November, the *European Journal of Psychotraumatology* published an interesting paper entitled “Treatment compliance and effectiveness in complex PTSD patients with co-morbid personality disorder undergoing stabilizing cognitive behavioral group treatment: a preliminary study” (Dorrepaal et al., 2013). This article describes a post hoc analysis on data derived from an analysis of a sample of complex PTSD patients previously published in the *Journal of Psychosomatics and Psychotherapy* (Dorrepaal et al., 2012). It shows the differential effects of an experimental condition of a psycho-educational and cognitive behavioral stabilizing group course added to treatment as usual (TAU) compared to TAU (only) for child-abuse-related complex PTSD. It appeared that drop-out in the stabilization course scarcely occurred in patients with the most severe (i.e., borderline) personality pathology (4%), whereas of those with fewer of such characteristics, 43% did not complete the course. We are concerned that the authors’ conclusions based upon their initial analysis, suggesting this program being efficacious (Dorrepaal et al., 2012), could prematurely encourage clinicians, as well as investigators, to offer their patients similar stabilization programs in affiliated mental health institutions. Therefore, we feel it as our obligation to share our alternate viewpoint with the readers.

The starting point for Dorrepaal's study (2012) was the widely recognized assumption (Cloitre et al., 2010), represented in a recent guideline of the International Society of Stress Studies (ISTSS; Cloitre et al., 2011), that patients suffering from complex PTSD are better prepared for trauma-memory processing by adding a first phase in which the primary focus is on stabilization by psycho-education, increasing affect regulation skills, and cognitive restructuring on trauma-related and interpersonal issues. Accordingly, Dorrepaal et al., using a randomized controlled trial, tested the efficacy of their stabilization course added to TAU among women with complex PTSD due to childhood sexual and physical abuse (Dorrepaal et al., 2012). To this end, patients were allocated to either 20 weekly 2-hour stabilization course meetings in addition to individual TAU, or to individual TAU only. General PTSD and complex PTSD symptoms decreased in both the stabilization course with TAU (large effect sizes) and the TAU-only condition (medium effect sizes). Based upon trends (0.05<*p*<0.10) with regard to some measures in the completers analysis, the authors concluded: *while significant superiority on change scores was absent, responder analysis suggested clinical meaningfulness of adding group treatment* (Dorrepaal et al., 2012, p. 1).

We question whether the authors drew the proper conclusion from the available data. The analytic strategy they choose to base their conclusion on, i.e., only evaluating the completers, is well known to be prone to bias (Fisher et al., 1990). Random allocation aims to ensure that trial participants’ risk factors that may affect the outcome under investigation are balanced between the allocated treatments. However, random allocation provides no guarantee that participants from each group, who do not comply with the allocated treatment, have the same risk-factor profile. That is the reason that intention-to-treat (ITT) analysis is widely recommended as the analysis of first choice (Hollis & Campbell, 1999). ITT compares the studied groups in terms of the treatment to which they were randomly allocated, irrespective of the treatment they actually received, protocol deviations and participant compliance or withdrawal from treatment. This is because in clinical practice, some patients are not fully compliant or drop out, and compliant subjects usually have better outcomes than noncompliant subjects. More specifically, in the case of a stabilization group program, good compliers might show stabilization benefits, poor compliers might have treatment responses similar to when they would not have learned affect regulation skills, whereas those who dropped out did not profit from the treatment at all. In other words, the trends in the Dorrepaal et al. primary analysis may have been an *overestimation* of the efficacy of the intervention due to the exclusion of the treatment responses of non-compliant participants in the (completers) analysis. Indeed, when the researchers applied ITT to their data, they did not find significant differences between the conditions. Accordingly—we think—they should have concluded that *the stabilizing group course did not have an additive gain over TAU*.

With regard to the efficacy of the stabilization course, the authors state that the pre-to-post effect size for this intervention in combination with TAU was large, and that for the TAU-only the effect size was medium. However, when one would subtract these effects to gain insight about the effectiveness of the stabilizing group course *per se*, the effect sizes may come close to what generally is considered as “placebo effects,” that could alternatively be explained as due to spontaneous improvement, symptom fluctuation, regression to the mean, or additional treatment (Linde, Fässler, & Meissner, 2011). With regard to the latter, although no information was provided regarding the number and length of the TAU sessions in both conditions, another paper that described a subgroup of the same sample, reported an average of 28 TAU sessions in the experimental condition (i.e., average hours of stabilization course and TAU combined: 28+40=68 hours, and 34 hours in the TAU only condition; Thomaes et al., 2012). Thus, patients in the experimental condition may have received twice as much treatment than suggested in the Dorrepaal et al., 2012 and 2013 articles.

Despite the fact that explicit trauma memory processing was not an ingredient of the stabilizing group course, the attrition rate was still relatively high (16% in Dorrepaal et al., 2012, p. 221, but reported as 18% in Dorrepaal et al., 2013, p. 4). In our opinion, the finding that one out of five patients drops out of an intervention lays the axe to the root of the phase-based approach, which is specifically designed to prepare patients for trauma memory processing, and to prevent them from dropping out prematurely. The explanation of the authors for this large drop-out rate was that stabilizing may not have been a good match for patients with less severe personality pathology. We agree with this view, but would like to add as a possible explanation that patients may have not seen the relevance, or the advantages of a lengthy intervention that does not directly address their traumatic memories. This hypothesis may also be applied to the group therapists, as it appeared that no less than 50% of them had to be replaced during the 20-week group intervention.

Taken together, in contrast to what Dorrepaal et al. (2012, 2013) suggest, we have serious doubts whether the stabilization course was as efficacious as suggested by the investigators. Furthermore, their findings are by no means supportive of the notion that patients with complex PTSD, or childhood abuse-related PTSD require a phase-based approach to prevent drop-out or to augment treatment outcome. Therefore, it is surprising that Dorrepaal et al. seem to present their findings as *support* for (“… in line with the rationale of a phased approach in Complex PTSD patients: stabilization if necessary; trauma focused if possible”; Dorrepaal et al., 2013, p. 5), rather than a *falsification of*, the phase-based hypothesis. But why the authors consider 40 hours (or more) spent on psycho-education, emotion regulation and cognitive restructuring, that added no effects on complex PTSD symptoms compared to TAU only, as being an *aid in providing optimal treatment to patients with child-abuse related (complex) PTSD* (Dorrepaal et al., 2013, p. 5) surprises even more.

*Ad De Jongh* Department of Behavioral Sciences ACTA, University of Amsterdam and VU University Amsterdam, The Netherlands School of Health Sciences Salford University Manchester, United Kingdom *Erik Ten Broeke* Visie, Private Practice for Cognitive Behavioural Therapy Deventer, The Netherlands
